# The Inhibition of Phosphoinositide-3 Kinases Induce Resolution of Inflammation in a Gout Model

**DOI:** 10.3389/fphar.2018.01505

**Published:** 2019-01-07

**Authors:** Izabela Galvão, Celso Martins Queiroz-Junior, Vivian Louise Soares de Oliveira, Vanessa Pinho, Emilio Hirsch, Mauro Martins Teixeira

**Affiliations:** ^1^Departamento de Bioquímica e Imunologia, Instituto de Ciências Biológicas, Universidade Federal de Minas Gerais, Belo Horizonte, Brazil; ^2^Departamento de Morfologia, Instituto de Ciências Biológicas, Universidade Federal de Minas Gerais, Belo Horizonte, Brazil; ^3^Department of Molecular Biotechnology and Health Sciences, University of Turin, Turin, Italy

**Keywords:** gout, neutrophil, resolution of inflammation, phosphoinositide-3 kinases, inflammation

## Abstract

Phosphoinositide-3 kinases (PI3Ks) are central signaling enzymes that are involved in many aspects of immune cell function. PI3Kγ and PI3Kδ are the major isoforms expressed in leukocytes. The role of PI3K isoforms in the resolution of inflammation is still poorly understood. Here, we investigated the contribution of PI3Kγ and PI3Kδ to the resolution of inflammation in a model of gout in mice.

**Methods and Results:** Experiments were performed in wild-type male C57/Bl6 mice. Selective inhibitors of PI3K-γ (AS605240) or PI3Kδ (GSK045) were injected in the joint 12 h after injection of MSU crystals, hence at the peak of inflammation. Inhibition of either PI3K isoform decreased number of neutrophils that migrated in response to the injection of MSU crystals. This was associated with reduction of myeloperoxidase activity and IL-1β levels in periarticular tissues and reduction of histological score. Joint dysfunction, as seen by reduced mechanical hypernociception, was improved by treatment with either inhibitor. The decrease in neutrophil numbers was associated with enhanced apoptosis and efferocytosis of these cells. There was shortening of resolution intervals, suggesting inhibition of either isoform induced the resolution of neutrophilic inflammation. Blockade of PI3Kγ or PI3Kδ reduced Nuclear Factor kappa B (NF-κB) activation. A pan-PI3K inhibitor (CL27c) reduced inflammation induced by MSU crystals by a magnitude that was similar to that attained by the PI3Kγ or PI3Kδ selective inhibitors alone.

**Conclusion:** Taken together, these results suggest that neutrophils can use PI3Kγ or PI3Kδ to remain in the cavity and blockade of either isoenzyme is sufficient to induce their apoptosis and resolve inflammation in a murine model of gout.

## Introduction

Gout is a disease caused by the deposition of monossodium urate (MSU) crystals in the joint and is characterized by swelling, redness, and intense pain. The prevalence and incidence of gout are increasing in both developed and developing countries (So and Martinon, 2017). Acute gouty inflammation is initiated by recognition of MSU crystals by resident cells that produce pro-inflammatory mediators, especially IL-1β that is released by activation of the Nucleotide-bindingdomain Leucine-Rich-containing family Pyrin domain-containing-3 (NLRP3) inflammasome (Martinon et al., 2006). IL-1β has a critical role in orchestrating the inflammatory reaction in response to MSU crystals and drives the production of chemokines and neutrophil influx into the joint (Amaral et al., 2012). Neutrophils are the main inflammatory cells recruited to the joint and contribute to the amplification of the inflammatory reaction, pain and progressive tissue damage (Busso and So, 2010). After migration, the lifespan of neutrophils is significantly extended under inflammatory conditions (Kolaczkowska and Kubes, 2013). Eventually, they undergo apoptosis and induce clearance by phagocytic macrophages in a process termed efferocytosis. Neutrophil apoptosis and subsequent efferocyosis constitute an important step in the resolution of inflammation and restoration of steady state (Serhan and Savill, 2005).

Resolution of inflammation, for many years, was considered a passive response, which was associated with the clearance of inflammatory stimulus, reduction of pro-inflammatory mediators and prevention of leukocyte recruitment. Resolution of inflammation is now considered an active process that involves synthesis of pro-resolving mediators that actively orchestrate the end of inflammation. In this context, the cardinal signs of resolution involves not only limitation of leukocyte migration and down regulation of chemokines and cytokines, but also the turning off of signaling pathways associated with leukocyte survival, which will eventually lead to leukocyte apoptosis and its subsequent efferocytosis (Sugimoto et al., 2016).

Phosphoinositide 3- kinase (PI3K) is a key regulator in signaling pathways triggered by a large numbers of receptors on the neutrophil surface (Hawkins et al., 2010). PI3Ks are enzymes that catalyze the phosphorylation of inositol phospholipidis in the third position of the inositol ring resulting in the formation of phosphatidylinositol-3-phosphate [PI(3)P], phosphatidylinositol 3,4-biphosphate [PI(3,4)P2], and phosphatidylinositol 3,4,5-trisphosphate [PI(3,4,5)P3], collectively termed 3′-PI lipids. There are three classes of PI3K: Class I isoforms: class IA (p110α, P110β, P110δ) and IB (PI3Kγ), Class II isoforms (PI3KC2α, β, and γ) and Class III a single isoform (Hawkins and Stephens, 2015). Isoforms PI3Kδ and PI3Kγ, from class I, are highly expressed in neutrophils suggesting that they have a particular role in these cells. PI3Ks are involved in neutrophil chemotaxis and it is also required for survival signals (Hawkins et al., 2010). Indeed, previous studies with non-isoform selective inhibitors – eg., Wortmaninn and LY294002 - have shown that blockade of PI3Ks is associated with induction of the resolution of inflammation in various models of inflammation (Pinho et al., 2005; Sousa et al., 2009).

In the current study, we investigated the effect of delayed inhibition of PI3Kγ or PI3Kδ, the major isoforms expressed in neutrophils, in a model of gout in mice. Our results show that blockade of PI3Ks induces resolution of inflammation by increasing neutrophil apoptosis and eferocytosis. Of interest, blockade of all PI3Ks or either PI3Kγ or PI3Kδ resulted in similar degree of inhibition suggesting that function of both PI3Kγ or PI3Kδ is necessary to guarantee neutrophil survival *in vivo*.

## Materials and Methods

### Mice

Male C57Bl/6 were obtained from the Center of bioterism of Universidade Federal de Minas Gerais (UFMG) Brazil. All mice were supplied with water and food *ad libitum*. Mice were maintained in pathogen free conditions. This study was carried out in accordance with the recommendations of the law n° 11.794 from National Council for Control of Animals Experimentation – CONCEA, Brazil. The protocol was approved by the Animal Ethics Council – CEUA – at Universidade Federal de Minas Gerais (protocol 2/2015). The total number of animals used in the current study was 130, distributed in experiments evaluating inhibition by selective PI3K inhibitor, for calculation of resolution indices, for histology, functional analysis (pain) and efferocytosis, as indicated in each relevant section of the manuscript.

### MSU Induced Gout

**FIGURE 1:**
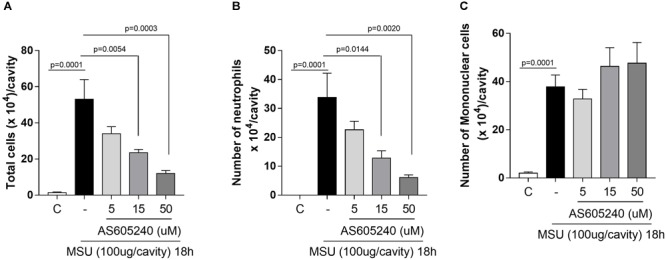
Dose response of PI3K-γ inhibitor in a MSU–induced gout inflammation. Mice were injected with MSU crystals (100 μg) into the tibiofemoral joint and the treatment with different doses of PI3K-γ inhibitor was made locally, 12 h after MSU injection. Cells were harvested from the articular cavity at 18 h after MSU injection. The number of total leukocytes **(A)** neutrophil **(B)**, and mononuclear cell **(C)** were evaluated. Results are expressed as the number of leukocytes per cavity and are shown as the mean ± SEM of five mice in each group from one experiment representative of two independent experiments. Significance was calculated using ANOVA followed by Holm-Sidak’s multiple comparison test. The exactly *p*-value are shown in the figure.

Crystals of MSU was prepared from uric acid (Sigma-Aldrich - St. Louis MO, United States) as previously described (Amaral et al., 2012). Mice under anesthesia (80:15 mg/kg ketamine:xylazine; i.p., Syntec, São Paulo, Brazil) were injected into the tibiofemoral knee joint with 100 μg of MSU crystals. The selective PI3K-γ inhibitor (AS605240 – Echelor), the selective PI3K-δ inhibitor [GSK045, (Gupta et al., 2016; Khan et al., 2017), kindly donated by GlaxoSmithKline - GSK], the PI3Kγ/δ inhibitor [CL27c, a pan-PI3K Inhibitor – (Pirali et al., 2017)] or vehicle were given locally (intraarticular injection) at 12 h after the injection of MSU crystals (see Figure 1). Inflammatory parameters were evaluated at different time points after treatment, as indicated in each figure legend. Mice were euthanized and the knee cavity washed with PBS/BSA 3% (2 × 5 μL) to collect the cells. The total number of leukocytes were determined using the newbauer chamber after staining with Turk’s solution. The differential counts were performed using standard morphologic criteria on a slide stained with May-Grunwald-Giemsa. Periarticular tissues were collected from the joints for evaluation of cytokines and myeloreproxidase (MPO) activity.

### Cytokines and Myeloperoxidase (MPO) Activity

Periarticular tissue was collected and homogenized in PBS containing anti-proteases, as previously described (Amaral et al., 2012). The concentration of IL-1β, CXCL1, TNF-α, and IL-10 was measured by ELISA assay in the supernatant of the homogenates and according to the instructions of the manufacturer (R&D systems). Results are expressed in pg/ml.

The myeloperoxidase activity assay was performed as previously described (Amaral et al., 2012). Briefly, the pellet from samples homogenized for cytokines measurements, were homogenized with 0.05M NaH_2_PO_4_ containing 0.5% of hexadecyltrimethyl-ammoniumbromide (HETAB; Signa-Aldrich). Samples were frozen 3 times in liquid nitrogen and centrifuged to collect the supernatant for MPO assay. The assay used 3,3′, 5,5′-tetramethylbenzidine (TMB; Sigma-Aldrich) and was quantified at 450 nm in a spectrophotometer. Results are expressed as absorbance.

### Evaluation of Hypernociception

The mechanical hypernociception were evaluated as previously described (Amaral et al., 2016) using an electronic pressure-meter (Insight instruments, Ribeirão Preto, SP, Brazil). The dorsiflexion-elicited withdrawal threshold was expressed in grams (g) and used to infer behavioral responses associated with experimental pain (hypernociception).

### Calculation of Resolution Indices

The resolution indices were quantified as previously described (Galvao et al., 2017). Briefly, cells were recovered from the knee lavage at 12, 18, 24, and 36 h after MSU challenge. The total cell count was determined using a Newbauer chamber and differential leukocyte counts using standard morphologic criteria on a slide stained with May-Grunwald-Giemsa. The Resolution interval (R*i*) was quantified by local kinetic of neutrophils infiltration defining the time interval between T*_max_* (peak of the infiltration) and T*_50_* (time when numbers of neutrophils drops to half of the peak).

### Histological Analysis

Samples were processed as previously described (Queiroz-Junior et al., 2011). Briefly, knee joints were collected, fixed in 10% formol and decalcified for 30 days in 14% EDTA. Tissues were included in paraffin, sectioned (5 μm) and stained with H&E. Two sections of knee joints were examined and scored by a pathologist (CQ-J) who was unaware of the experimental groups. The parameters evaluated were: severity of synovial hyperplasia, intensity and extension of inflammatory infiltrate, vascular hyperemia, presence of inflammatory cells in the synovial cavity and changes in tissue architecture. These criteria ranged from 0 to 8 points and the sum was used to obtain a histological score.

### Western Blot Analysis

Synovial tissue samples (20–40 mg tissue) were homogenized using a cell lysis buffer, as described (Galvao et al., 2017). Protein amounts were quantified with the Bradford assay reagent from Bio-Rad (Hercules, CA, United States). Extracts (40 μg) were separated by electrophoresis on a denaturing, 10% polyacrylamide-SDS gel and electrotransferred to nitrocellulose membranes. Membranes was incubated with specific primary antibodies (anti-IκBα – Cell Signaling) and then incubated with appropriated HRP-conjugated secondary antibody. Immunoreactive bands were visualized by using an ECL detection system, as described by the manufacturer (GE Healthcare, Piscataway, NJ, United States). For loading control, membranes were reprobed with anti-GAPDH (Sigma).

### Assessment of Apoptosis and Efferocytosis

Apoptosis and efferocytosis were assessed by flow cytometry, as described previously (Dalli et al., 2013). Mice were injected with MSU crystals and 12 h later they were treated locally with PI3K inhibitors. For the apoptosis assays, the lavage of the knee was performed 4 h after the treatment with drugs. Cells were surface-stained for 30 min with anti-LY6G-BV421 antibody (eBioscience) and then labeled with annexin-V FITC and PI, as an index of loss of nuclear membrane integrity (PE Annexin V Apoptosis Detection Kit; BD PharmingenTM; United States). For the efferocytosis assays, joint wasy was performed 6 h after the treatment with PI3K inhibitors and cells were surface-stained for 30 min with anti-F4/80-PECy7 antibody (eBioscience). Then, cells were fixed for 10 min, treated with 1× permeabilization wash (Cytofix/Cytoperm Kit; BD Biosciences) and intracellularly stained with anti-Ly6G-BV421 antibody. Macrophage efferocytosis was assessed as a frequency of macrophages containing neutrophils (F4/80+ Ly6G+ cells).

### Statistical Analysis

The number of animals used in this study were determined by using a statistical software (GPOWER 3.1.9.2) during the preparation of the study design, considering the variation of numbers of neutrophils in the knee lavage in our previous publications. All results are presented as the mean ± SEM. The analysis of the difference between two groups was performed by two-tailed unpaired Student’s T test. Normalized data were analyzed by one-way ANOVA, and differences between groups were assessed using the Holm-Sidak’s multiple comparison *post hoc* test. A *p*-value < 0.05 was considered significant. Calculations were performed using the Prism 7.0 software program for Windows (GraphPad software, San Diego, CA, United States).

## Results

### PI3K-γ Inhibitor Reverses Neutrophils Recruitment in a Dose-Dependent Manner

As previously reported, MSU crystals induced influx of leukocytes, mainly neutrophils, into the knee joint at 18 h after injection. The treatment with a specific PI3K-γ inhibitor, AS605240, at the peak of the inflammation (i.e., 12 h after administration of MSU crystals) was efficient to reduce the number of accumulated neutrophils in a dose dependent manner (Figure 1B). The highest dose caused the greatest reduction of the number of neutrophils in the cavity and was used for further experiments. No difference was observed in the number of infiltrating mononuclear cells (Figure 1C).

### Delayed Inhibition of PI3K-γ or PI3K-δ Induce Timely Resolution of Neutrophilic Inflammation

**FIGURE 2:**
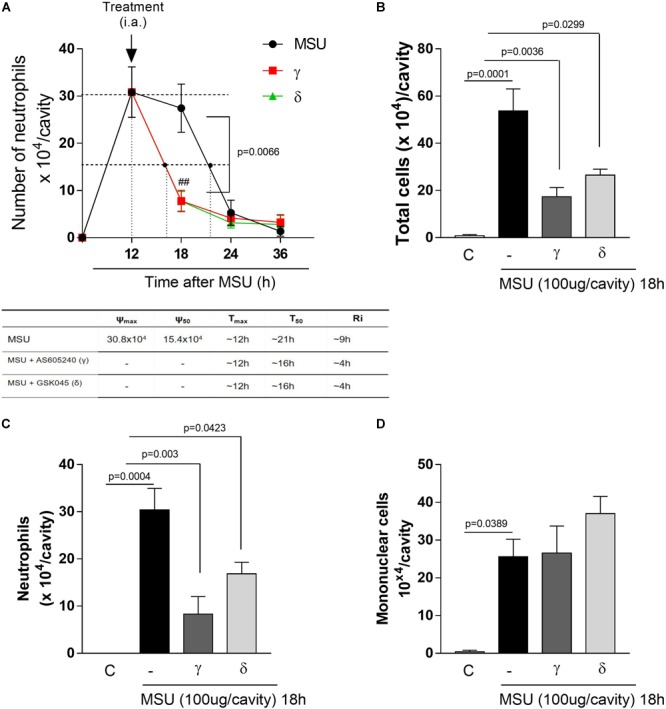
Delayed treatment of PI3K-γ or PI3K-δ inhibitor in a MSU–induced gout inflammation induce timely resolution. Mice were injected with MSU crystals (100 μg) into the tibiofemoral joint and the treatment with intraarticular injection (i.a.,) of 50 μM of PI3K-γ or PI3K-δ inhibitor, 12 h after MSU injection. Cells were harvested from the articular cavity at 18, 24, and 36 h after MSU injection. The number of neutrophils and resolution indices were quantified **(A)**. Of note: ψ_max_ = maximal number of neutrophils, ψ_50_ = 50% of the maximum number of neutrophils, T_max_ = 12 h, the time point when neutrophil numbers reach maximum; T_50_ MSU+AS605240 and MSU+GSK045 group ∼ 16 h, the time point when PMN numbers reduce to 50% of maximum; and resolution interval R_i_ MSU+AS605240 and MSU+GSK045 group ∼ 4 h, the time period when 50% PMN are lost from the articular cavity. Leukocytes counts 18 h after MSU injection **(B)** total leukocytes numbers, **(C)** neutrophils, **(D)** mononuclear cells. Results are expressed as the number of leukocytes per cavity and are shown as the mean ± SEM of five mice in each group from one experiment representative of two independent experiments. Significance was calculated using ANOVA followed by Holm-Sidak’s multiple comparison test. The exactly *p*-value are shown in the figure. ^##^means *p* value < 0.01 compared with 18 h MSU injected.

To determine the effects of the drugs on the kinetics of neutrophil infiltration, we calculated the resolution indices (R*i*) after drug treatment. AS605240 or GSK045, a PI3K-δ inhibitor, were injected intraarticular at the peak of acute inflammation (i.e., 12 h) and cells from the synovial cavity harvested 6, 12, and 24 h after the drugs were given. The treatment with AS605240 or GSK045 shortened the resolution interval by ∼5h. These resolution indices R*i*: R*i*_MSU_: ∼9 h; R*i*_MSU+AS605240_: ∼4 h; R*i*_MSU+GSK045_: ∼4 h (Figure 2A) suggests an acceleration in the resolution of acute inflammation. At 18 h after injection of MSU crystals (6 h after injection of PI3K inhibitors), there was significant reduction of the number of total leukocytes (Figure 2B) and this reduction was due to inhibition of neutrophils (Figure 2C), but not mononuclear cells (Figure 2D). Overall, treatment with either AS605240 or GSK045 resulted in similar degree of inhibition of the parameters observed.

### Inhibition of PI3K-γ or PI3K-δ Reduced Neutrophil Accumulation and Cytokines That Mediates Joint Inflammation

**FIGURE 3:**
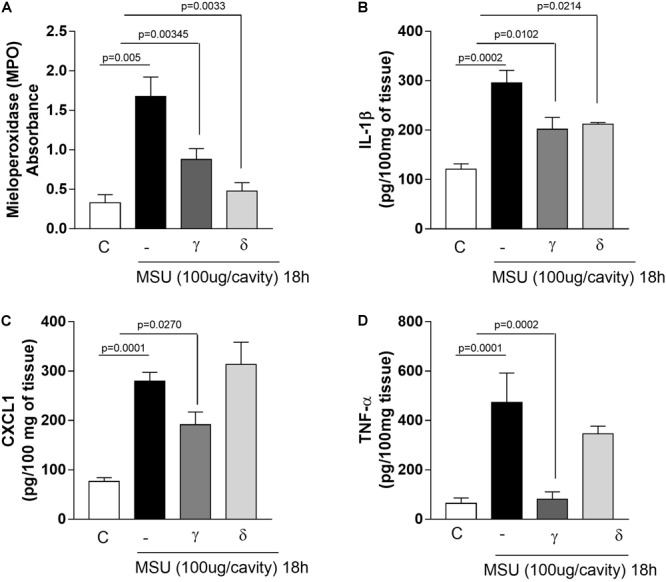
Effects of PI3K-γ or PI3K-δ inhibition on neutrophil accumulation and pro-inflammatory mediators production. Myeloperoxidase activity in homogenized periarticular tissue **(A)**. IL-1β levels **(B)**, CXCL1 **(C)**, and TNF-α levels **(D)**. Levels was measured by ELISA in supernatant of homogenized periarticular tissue. Results are expressed as the number of leukocytes per cavity and are shown as the mean ± SEM of five mice in each group from one experiment representative of two independent experiments. Significance was calculated using ANOVA followed by Holm-Sidak’s multiple comparison test. The exactly *p*-value are shown in the figure.

To evaluate the inflammatory response after inhibition of different PI3K isoforms, we investigated the accumulation of neutrophils and production of pro-inflammatory cytokines in the periarticular tissue of mice 18 h after injection of MSU crystals. Both treatments reduced to a similar extent the accumulation of neutrophils in periarticular tissues, as assessed by measuring MPO activity (Figure 3A). Injection of MSU crystals induced an increase in levels of the pro-inflammatory mediators IL-1β, CXCL1, and TNF-α in periarticular tissue. Overall, the treatment with the PI3K-γ or PI3K-δ inhibitors caused a reduction of levels of pro-inflammatory mediators but the effects of the PI3K-γ inhibitor was more prominent than that of the PI3K-δ inhibitor (Figures 3B–D).

### Inhibition of PI3K-γ or PI3K-δ Ameliorated Tissue Damage and Mechanical Hypernociception

**FIGURE 4:**
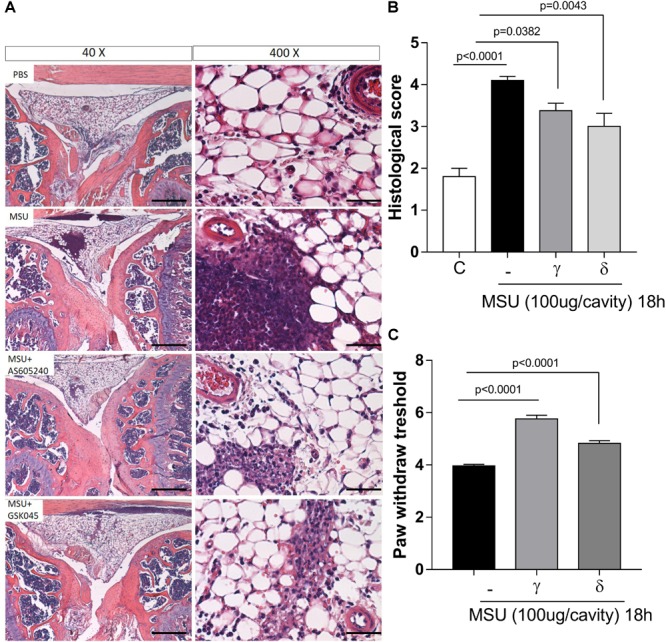
Effects of delayed treatment of PI3K-γ or PI3K-δ inhibitor on tissue damage and hypernociceptive responses. Representative photographs of H&E-stained sections of knee from mice control (PBS), MSU challenge (MSU), mice challenge with MSU and post treated (12 h) with AS605240 (MSU+AS) and mice challenge with MSU and post treated (12 h) with GSK045 (MSU+GSK045) (bars represent 400 μm in magnification of 40× and 50 μm in magnification of 400×) **(A)**. Graph shown histological score of joint injury of MSU crystal injected mice **(B)**. Mechanical hypernociception was evaluated by an electronic pressure-meter 18 h after the injection of MSU crystals (100 μg; ia) treated or not with AS605240 or GSK045 (γ or δ – 50 μM i.a.) **(C)**. Results are expressed as the number of leukocytes per cavity and are shown as the mean ± SEM of five mice in each group from one experiment representative of two independent experiments. Significance was calculated using ANOVA followed by Holm-Sidak’s multiple comparison test. The exactly *p*-value are shown in the figure.

After having demonstrated the pro-resolving properties of PI3K-γ or PI3K-δ inhibition, we turned our attention to the possible impact of treatment on tissue damage. Histological analysis of knees subjected to intraarticular injection of MSU crystals showed moderate infiltration of leukocytes, focal hyperplasia, and leukocytes in the synovial space 18 h after MSU crystal injection. Inhibition of PI3K-γ or PI3K-δ decreased all the observed parameters (Figure 4A) and resulted in overall decrease of the histopathological score (Figure 4B).

Injection of MSU crystals induces mechanical hypernociception, an index of joint dysfunction, as measured by decrease in paw withdrawal threshold. The intraarticular injection of the PI3K-γ or PI3K-δ inhibitors reduced to a similar extent the hypernociception induced by the injection of MSU crystals (Figure 4C).

### Mechanistically, the Inhibition of PI3K-γ or PI3K-δ Induced Neutrophils Apoptosis and Efferocytosis

**FIGURE 5:**
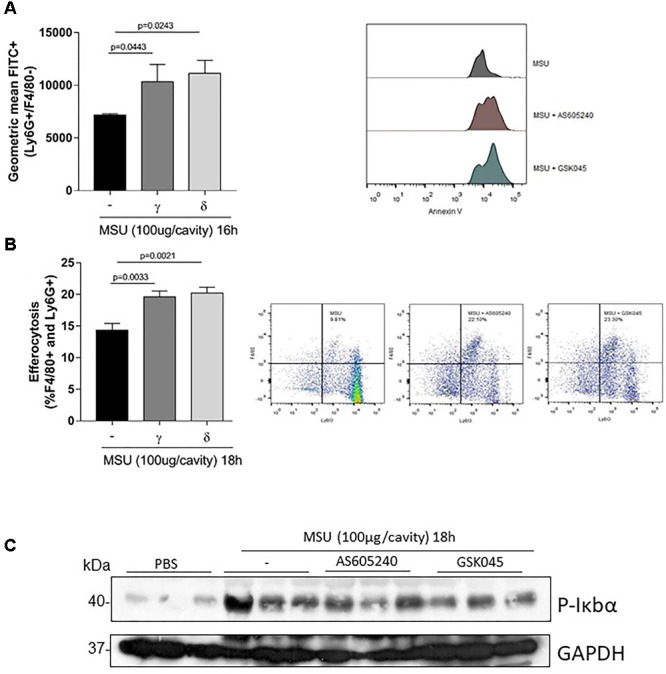
Effect of PI3K-γ inhibition or PI3K-δ inhibition on neutrophil apoptosis and efferocytosis in MSU-induced inflammation. Mice were injected with MSU crystals (100 μg) into the tibiofemoral joint and 12 h later received an injection of AS605240 or GSK045 (50 μM, i.a.). Four hours after treatment, the cells were collected to annexin V analysis **(A)**. Efferocytosis was evaluated in mice injected with MSU crystals (100 μg) into the tibiofemoral joint and 12 h later received an injection of AS605240 or GSK045. Knee was washed 18 h MSU injection and cells were surface-stained with anti-F4/80 for macrophages and then intracellularly stained with anti-Ly6G for neutrophils **(B)**. Results are expressed as the number of leukocytes per cavity and are shown as the mean ± SEM of five mice in each group from one experiment representative of two independent experiments. Significance was calculated using ANOVA followed by Holm-Sidak’s multiple comparison test. The exactly *p*-value are shown in the figure. **(C)** Expression of p-IκBα by Western Blot in synovial tissue collected 18 h after MSU injection. For loading control, membrane was reprobed with anti-GAPDH.

Next, we investigated whether induction of neutrophil apoptosis and subsequent efferocytosis could account for the resolution of MSU crystal-induced inflammation. For this purpose, mice were treated intraarticularly with inhibitors at the peak of inflammation and cells harvested from the knee cavity 4 h after the treatment to evaluate apoptosis and 6 h after the treatment to evaluate efferocytosis. We observed that treatment with either inhibitor increased neutrophil apoptosis and efferocytosis to a similar extent (Figures 5A,B). Noteworthy, these events were associated with reduction in phosphorylation of IκBα, an important regulator of the pro-survival molecule NFκB (Figure 5C) (Original western blot Supplementary Figure S1). Taken together, ours results clearly suggest that inhibition of PI3K isoforms γ or δ accelerate apoptosis and efferocytosis through down-modulation of NFκB leading to resolution of inflammation.

### Pan-PI3K Inhibition Produced the Same Effects of Isolated Inhibition

**FIGURE 6:**
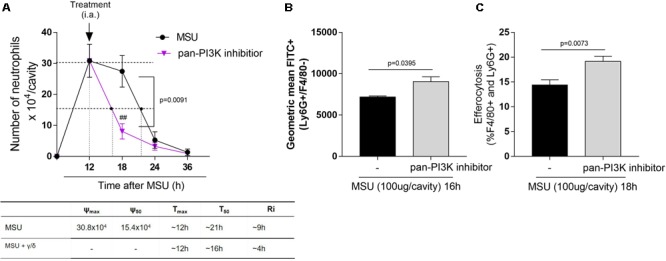
Effect of pan-PI3K inhibition on neutrophil recruitment, apoptosis and efferocytosis in MSU-induced inflammation. Mice were injected with MSU crystals (100 μg) into the tibiofemoral joint and the treatment with intraarticular injection (i.a.) of 50 μM of pan-PI3K inhibitor, 12 h after MSU injection. Cells were harvested from the articular cavity at 18, 24, and 36 h after MSU injection. The number of neutrophils and resolution indices were quantified (A). Of note: ψ_max_ = maximal number of neutrophils, ψ_50_ = 50% of the maximum number of neutrophils, T_max_ = 12 h, the time point when neutrophil numbers reach maximum; T_50_ MSU+pan-PI3K inhibitor group ∼ 16 h, the time point when PMN numbers reduce to 50% of maximum; and resolution interval R_i_ MSU+pan-PI3K inhibitor group (γ/δ) ∼ 4 h, the time period when 50% PMN are lost from the articular cavity. **(A)** Apoptosis assay was evaluated 4 h after treatment, the cells were collected to annexin V analysis **(B)**. Efferocytosis was evaluated in mice injected with MSU crystals (100 μg) into the tibiofemoral joint and 12 h later received an injection of pan-PI3K inhibitor. Cells were collected 18 h after MSU crystal injection and surface-stained with anti-F4/80 for macrophages and then intracellularly stained with anti-Ly6G for neutrophils **(C)**. Results are shown as the mean ± SEM of five mice in each group and are from one experiment representative of two independent experiments. Significance was calculated in relation to the control group (two-tailed unpaired Student’s *t*-test). The exactly *p*-value are shown in the figure. ^##^means *p* value < 0.01 compared with 18 h MSU injected.

A pan-PI3K inhibitor, CL27c, was used to investigate whether inhibition of both PI3K isoforms could cooperate to achieve greater induction of resolution of inflammation. The pan-PI3K inhibition shortened the resolution interval by ∼5h, a result similar to that achieved by inhibiting either enzyme alone (Figure 6A). Noteworthy, the pan-PI3K inhibitor also induced apoptosis and subsequent efferocytosis of neutrophils recruited to the cavity (Figures 6B,C). Again, these effects were similar in magnitude to those observed with treatment with either PI3K inhibitor applied alone.

## Discussion

The major findings of the current study were: (i) delayed inhibition of either PI3K-γ or PI3K-δ led to reduction of the accumulation of neutrophils in the joint cavity in response to the injection of MSU crystals. Reduction of neutrophil numbers was associated with reduction in the levels of pro-inflammatory mediators, decreased hypernociception and of tissue damage induced by MSU crystals. (iii) The beneficial effects of the delayed treatment with either inhibitor was secondary to their ability to cause resolution of inflammation, as seen by the induction of neutrophil apoptosis, their subsequent eferocytosis and faster restoration of steady state in the cavity (Martinon et al., 2006). Inhibition of either PI3K isoform was sufficient to attain full inhibition of most inflammatory parameters. Indeed, there was no additional resolution of inflammation when a pan-PI3K inhibitor was used.

Inhibition of phosphatidylinositol 3-kinases (PI3Ks) have been shown to inhibit the recruitment of neutrophils in various models of inflammation (Ghigo et al., 2010). For example, blockade of PI3K-γ using AS605240 supressed joint inflammation and damage in a murine model of rheumatoid arthritis (Camps et al., 2005). Similarly, pharmacological blockade of PI3K-δ in K/BxN serum transfer model of arthritis reduced the overall extent of inflammation (Randis et al., 2008). We have shown that inhibition of both PI3K-γ and PI3K-δ was necessary for prevention of the recruitment of neutrophils *in vivo*, showing these enzymes played a redundant role in CXCL1-mediated neutrophil influx (Pinho et al., 2007). Other studies have shown that non-selective inhibition of PI3K isoforms induced resolution of inflammation (Pinho et al., 2005; Sousa et al., 2009). Different from anti-inflammatory therapies that control inflammation by blocking key pro-inflammatory mediators that are expressed in the initial phase of the inflammation, pro-resolving therapies involve modifying the course of an established inflammation by reducing the time and accelerating the resolution (Serhan et al., 2007; Fullerton and Gilroy, 2016). Here, we show for the first time that delayed treatment with selective inhibitors for PI3K-γ or PI3K-δ affected the resolution of inflammation, by reducing the time necessary to decrease 50% of neutrophil from the maximal number, demonstrating the potential pro-resolving properties of selective PI3K inhibition.

Inhibition of class Ia PI3K, with non-selective inhibitors, potentially downregulate cytokines generation in neutrophils (Fortin et al., 2011). The interaction of MSU crystals with neutrophil leads to activation of class Ia PI3K that is involved in neutrophil degranulation (Popa-Nita et al., 2007). In gout, neutrophils are largely responsible for tissue damage caused by excessive release of granules into the synovial fluid (Dalbeth and Haskard, 2005). In addition to tissue damage, pain experienced by gouty patients during acute gout attack are the single most common reasons for these patients to seek medical care (Ruiz-Miyazawa et al., 2018). Mechanical hypernociceptive response, an index of inflammatory pain, is associated with high number of neutrophils and increased levels of IL-1β (Amaral et al., 2012; Galvao et al., 2017). Here, treatment with inhibitors of either isoforms was sufficient to reduce the secretion of pro-inflammatory cytokines, and this was associated with reduced number of neutrophils in the periarticular tissue (MPO), reduced tissue damage and pain associated with MSU crystal-induced inflammation.

Previous studies have shown that non-selective PI3K inhibitors induced resolution of inflammation by increasing apoptosis of eosinophils (Pinho et al., 2005; Sousa et al., 2009) and neutrophils (Lopes et al., 2011) in sites of inflammation induced by various stimuli. The clearance of apoptotic granulocytes is mostly mediated by efferocytosis in inflamed tissue (Greenlee-Wacker, 2016). Here, we clearly show that selective blockade of PI3K-γ or PI3K-δ was sufficient to induce effectively apoptosis of neutrophils and induced their subsequent efferocytosis in a murine model of gout. Together with the reduction in resolution intervals, our results clearly show that inhibition of either enzyme will induce resolution of inflammation, suggesting that neutrophils may use PI3K-γ or PI3K-δ to remain at sites of inflammation *in vivo*.

Induction of the resolution of inflammation by treatment with a pan-PI3K inhibitor was similar in magnitude to that attained with either PI3K-γ or PI3K-δ inhibition separately. These results concur with the idea that neutrophils can use either PI3K-γ or PI3K-δ to remain at sites of inflammation. NF-κB appears to be a major transcription factor involved in the persistence of neutrophils at sites of inflammation (Nathan, 2002; Lawrence, 2009). Indeed, we have shown that blockade of NF-κB resolve inflammation in various models of inflammation (Vago et al., 2015, 2016; Barroso et al., 2017), including in a model of gout (Vieira et al., 2017). Other studies have shown that lipoxin A4 may modulate neutrophil infiltration by reducing the activation of NF-κB (Devchand et al., 2003, 2005). Importantly, non-selective inhibition of PI3K decreased NF-κB p65 translocation to the nucleus, suggesting that NF-κB is downstream of PI3K (Lopes et al., 2011). Our results clearly show that inhibition of either PI3K-γ or PI3K-δ reduced activation of NF-κB in synovial tissues. Therefore, it appears that a major mechanism by which inhibition of PI3K controls neutrophil persistence in tissues is via control of NF-κB activation.

Taken together, our results show that inhibition of PI3K-γ or PI3K-δ isoforms induce resolution of inflammation in a murine model of gout. There was characterized by neutrophil apoptosis and increased efferocytosis and consequent reduction of pro-inflammatory mediators, hypernociception and tissue damage. It is unclear why both PI3K-γ and PI3K-δ are necessary to maintain neutrophil survival in the cavity; i.e., Blockade of either enzyme is sufficient to attain faster resolution of inflammation. Moreover, it is unclear whether blockade of these enzymes will translate into resolution of inflammation in more complex and chronic models of inflammation. Regardless of limitation, our results clearly show that PI3K-γ or PI3K-δ are needed to maintain neutrophil persistence in inflammatory infiltrates.

## Author Contributions

IG, VP, EH, and MT designed the research and wrote the manuscript. IG performed the experiments and analyzed the data. CQ-J performed the histological analysis. VdO performed the hypernociception evaluation. EH synthesized the pan-PI3K inhibitor.

## Conflict of Interest Statement

The authors declare that the research was conducted in the absence of any commercial or financial relationships that could be construed as a potential conflict of interest.

## Supplementary Material

FIGURE S1 Original western blot from the expression of p-IκBα in synovial tissue collected 18 h after MSU injection. For loading control, membrane was reprobed with anti-GAPDH.

## References

[B1] Amaral F. A. Bastos L. F. Oliveira T. H. Dias A. C. Oliveira V. L. Tavares L. D. (2016). Transmembrane TNF-alpha is sufficient for articular inflammation and hypernociception in a mouse model of gout. *Eur. J. Immunol.* 46 204–211. 10.1002/eji.201545798 26449770

[B2] Amaral F. A. Costa V. V. Tavares L. D. Sachs D. Coelho F. M. Fagundes C. T. (2012). NLRP3 inflammasome-mediated neutrophil recruitment and hypernociception depend on leukotriene B(4) in a murine model of gout. *Arthritis Rheum.* 64 474–484. 10.1002/art.33355 21952942

[B3] Barroso L. C. Magalhaes G. S. Galvao I. Reis A. C. Souza D. G. Sousa L. P. (2017). Angiotensin-(1-7) promotes resolution of neutrophilic inflammation in a model of antigen-induced arthritis in mice. *Front. Immunol.* 8:1596. 10.3389/fimmu.2017.01596 29209329 PMC5701946

[B4] Busso N. So A. (2010). Mechanisms of inflammation in gout. *Arthritis Res. Ther.* 12:206. 10.1186/ar2952 20441605 PMC2888190

[B5] Camps M. Ruckle T. Ji H. Ardissone V. Rintelen F. Shaw J. (2005). Blockade of PI3Kgamma suppresses joint inflammation and damage in mouse models of rheumatoid arthritis. *Nat. Med.* 11 936–943. 10.1038/nm1284 16127437

[B6] Dalbeth N. Haskard D. O. (2005). Mechanisms of inflammation in gout. *Rheumatology* 44 1090–1096. 10.1093/rheumatology/keh640 15956094

[B7] Dalli J. Consalvo A. P. Ray V. Di Filippo C. D’amico M. Mehta N. (2013). Proresolving and tissue-protective actions of annexin A1-based cleavage-resistant peptides are mediated by formyl peptide receptor 2/lipoxin A4 receptor. *J. Immunol.* 190 6478–6487. 10.4049/jimmunol.1203000 23686496

[B8] Devchand P. R. Arita M. Hong S. Bannenberg G. Moussignac R. L. Gronert K. (2003). Human ALX receptor regulates neutrophil recruitment in transgenic mice: roles in inflammation and host defense. *FASEB J.* 17 652–659. 10.1096/fj.02-0770com 12665478

[B9] Devchand P. R. Schmidt B. A. Primo V. C. Zhang Q. Y. Arnaout M. A. Serhan C. N. (2005). A synthetic eicosanoid LX-mimetic unravels host-donor interactions in allogeneic BMT-induced GvHD to reveal an early protective role for host neutrophils. *FASEB J.* 19 203–210. 10.1096/fj.04-2565com 15677343

[B10] Fortin C. F. Cloutier A. Ear T. Sylvain-Prevost S. Mayer T. Z. Bouchelaghem R. (2011). A class IA PI3K controls inflammatory cytokine production in human neutrophils. *Eur. J. Immunol.* 41 1709–1719. 10.1002/eji.201040945 21469098

[B11] Fullerton J. N. Gilroy D. W. (2016). Resolution of inflammation: a new therapeutic frontier. *Nat. Rev. Drug Discov.* 15 551–567. 10.1038/nrd.2016.39 27020098

[B12] Galvao I. Vago J. P. Barroso L. C. Tavares L. P. Queiroz-Junior C. M. Costa V. V. (2017). Annexin A1 promotes timely resolution of inflammation in murine gout. *Eur. J. Immunol.* 47 585–596. 10.1002/eji.201646551 27995621

[B13] Ghigo A. Damilano F. Braccini L. Hirsch E. (2010). PI3K inhibition in inflammation: toward tailored therapies for specific diseases. *Bioessays* 32 185–196. 10.1002/bies.200900150 20162662

[B14] Greenlee-Wacker M. C. (2016). Clearance of apoptotic neutrophils and resolution of inflammation. *Immunol. Rev.* 273 357–370. 10.1111/imr.12453 27558346 PMC5000862

[B15] Gupta V. Khan A. Higham A. Lemon J. Sriskantharajah S. Amour A. (2016). The effect of phosphatidylinositol-3 kinase inhibition on matrix metalloproteinase-9 and reactive oxygen species release from chronic obstructive pulmonary disease neutrophils. *Int. Immunopharmacol.* 35 155–162. 10.1016/j.intimp.2016.03.027 27049289

[B16] Hawkins P. T. Stephens L. R. (2015). PI3K signalling in inflammation. *Biochim. Biophys. Acta* 1851 882–897. 10.1016/j.bbalip.2014.12.006 25514767

[B17] Hawkins P. T. Stephens L. R. Suire S. Wilson M. (2010). PI3K signaling in neutrophils. *Curr. Top. Microbiol. Immunol.* 346 183–202. 10.1007/82_2010_40 20473789

[B18] Khan A. Southworth T. Worsley S. Sriskantharajah S. Amour A. Hessel E. M. (2017). An investigation of the anti-inflammatory effects and a potential biomarker of PI3Kdelta inhibition in COPD T cells. *Clin. Exp. Pharmacol. Physiol.* 44 932–940. 10.1111/1440-1681.12784 28508433

[B19] Kolaczkowska E. Kubes P. (2013). Neutrophil recruitment and function in health and inflammation. *Nat. Rev. Immunol.* 13 159–175. 10.1038/nri3399 23435331

[B20] Lawrence T. (2009). The nuclear factor NF-kappaB pathway in inflammation. *Cold Spring Harb. Perspect. Biol.* 1:a001651. 10.1101/cshperspect.a001651 20457564 PMC2882124

[B21] Lopes F. Coelho F. M. Costa V. V. Vieira E. L. Sousa L. P. Silva T. A. (2011). Resolution of neutrophilic inflammation by H2O2 in antigen-induced arthritis. *Arthritis Rheum.* 63 2651–2660. 10.1002/art.30448 21567381

[B22] Martinon F. Petrilli V. Mayor A. Tardivel A. Tschopp J. (2006). Gout-associated uric acid crystals activate the NALP3 inflammasome. *Nature* 440 237–241. 10.1038/nature04516 16407889

[B23] Nathan C. (2002). Points of control in inflammation. *Nature* 420 846–852. 10.1038/nature01320 12490957

[B24] Pinho V. Russo R. C. Amaral F. A. De Sousa L. P. Barsante M. M. De Souza D. G. (2007). Tissue- and stimulus-dependent role of phosphatidylinositol 3-kinase isoforms for neutrophil recruitment induced by chemoattractants in vivo. *J. Immunol.* 179 7891–7898. 10.4049/jimmunol.179.11.7891 18025236

[B25] Pinho V. Souza D. G. Barsante M. M. Hamer F. P. De Freitas M. S. Rossi A. G. (2005). Phosphoinositide-3 kinases critically regulate the recruitment and survival of eosinophils in vivo: importance for the resolution of allergic inflammation. *J. Leukoc. Biol.* 77 800–810. 10.1189/jlb.0704386 15860799

[B26] Pirali T. Ciraolo E. Aprile S. Massarotti A. Berndt A. Griglio A. (2017). Identification of a potent phosphoinositide 3-Kinase pan inhibitor displaying a strategic carboxylic acid group and development of its prodrugs. *ChemMedChem* 12 1542–1554. 10.1002/cmdc.201700340 28857471 PMC5697638

[B27] Popa-Nita O. Rollet-Labelle E. Thibault N. Gilbert C. Bourgoin S. G. Naccache P. H. (2007). Crystal-induced neutrophil activation. IX. Syk-dependent activation of class Ia phosphatidylinositol 3-kinase. *J. Leukoc. Biol.* 82 763–773. 10.1189/jlb.0307174 17535983

[B28] Queiroz-Junior C. M. Madeira M. F. Coelho F. M. Costa V. V. Bessoni R. L. Sousa L. F. (2011). Experimental arthritis triggers periodontal disease in mice: involvement of TNF-alpha and the oral microbiota. *J. Immunol.* 187 3821–3830. 10.4049/jimmunol.1101195 21890656

[B29] Randis T. M. Puri K. D. Zhou H. Diacovo T. G. (2008). Role of PI3Kdelta and PI3Kgamma in inflammatory arthritis and tissue localization of neutrophils. *Eur. J. Immunol.* 38 1215–1224. 10.1002/eji.200838266 18412166 PMC2972192

[B30] Ruiz-Miyazawa K. W. Staurengo-Ferrari L. Pinho-Ribeiro F. A. Fattori V. Zaninelli T. H. Badaro-Garcia S. (2018). 15d-PGJ2-loaded nanocapsules ameliorate experimental gout arthritis by reducing pain and inflammation in a PPAR-gamma-sensitive manner in mice. *Sci. Rep.* 8:13979. 10.1038/s41598-018-32334-0 30228306 PMC6143605

[B31] Serhan C. N. Brain S. D. Buckley C. D. Gilroy D. W. Haslett C. O’neill L. A. (2007). Resolution of inflammation: state of the art, definitions and terms. *FASEB J.* 21 325–332. 10.1096/fj.06-7227rev 17267386 PMC3119634

[B32] Serhan C. N. Savill J. (2005). Resolution of inflammation: the beginning programs the end. *Nat. Immunol.* 6 1191–1197. 10.1038/ni1276 16369558

[B33] So A. K. Martinon F. (2017). Inflammation in gout: mechanisms and therapeutic targets. *Nat. Rev. Rheumatol.* 13 639–647. 10.1038/nrrheum.2017.155 28959043

[B34] Sousa L. P. Carmo A. F. Rezende B. M. Lopes F. Silva D. M. Alessandri A. L. (2009). Cyclic AMP enhances resolution of allergic pleurisy by promoting inflammatory cell apoptosis via inhibition of PI3K/Akt and NF-kappaB. *Biochem. Pharmacol.* 78 396–405. 10.1016/j.bcp.2009.04.030 19422809

[B35] Sugimoto M. A. Sousa L. P. Pinho V. Perretti M. Teixeira M. M. (2016). Resolution of inflammation: what controls its onset? *Front. Immunol.* 7:160. 10.3389/fimmu.2016.00160 27199985 PMC4845539

[B36] Vago J. P. Tavares L. P. Garcia C. C. Lima K. M. Perucci L. O. Vieira E. L. (2015). The role and effects of glucocorticoid-induced leucine zipper in the context of inflammation resolution. *J. Immunol.* 194 4940–4950. 10.4049/jimmunol.1401722 25876761

[B37] Vago J. P. Tavares L. P. Sugimoto M. A. Lima G. L. Galvao I. De Caux T. R. (2016). Proresolving actions of synthetic and natural protease inhibitors are mediated by annexin A1. *J. Immunol.* 196 1922–1932. 10.4049/jimmunol.1500886 26800869

[B38] Vieira A. T. Galvao I. Macia L. M. Sernaglia E. M. Vinolo M. A. Garcia C. C. (2017). Dietary fiber and the short-chain fatty acid acetate promote resolution of neutrophilic inflammation in a model of gout in mice. *J. Leukoc. Biol.* 101 275–284. 10.1189/jlb.3A1015-453RRR 27496979

