# Supplementary figures and images for: The Inhibition of Phosphoinositide-3 Kinases Induce Resolution of Inflammation in a Gout Model
Source: Front Pharmacol. 2019 Jan 7;9:1505. doi: 10.3389/fphar.2018.01505 (PMC6330337; doi:10.3389/fphar.2018.01505)

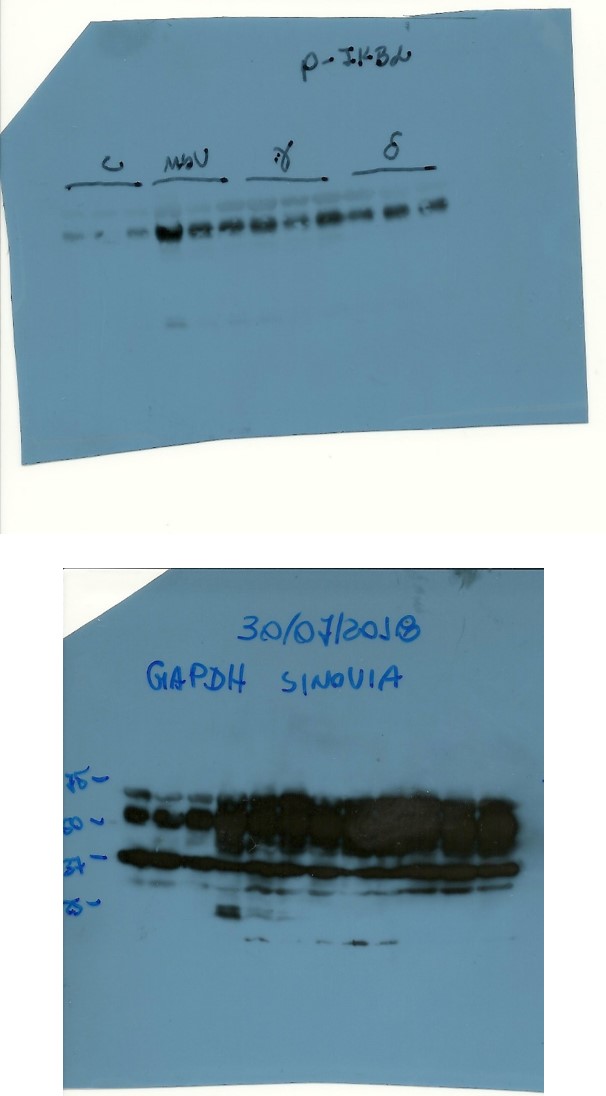

Supplement: FIGURE S1 — Original western blot from the expression of p-IκBα in synovial tissue collected 18 h after MSU injection. For loading control, membrane was reprobed with anti-GAPDH. [file Image_1.JPEG]
